# Advanced HIV disease management practices within inpatient medicine units at a referral hospital in Zambia: a retrospective chart review

**DOI:** 10.1186/s12981-022-00433-8

**Published:** 2022-02-22

**Authors:** Nyuma Mbewe, Michael J. Vinikoor, Sombo Fwoloshi, Mundia Mwitumwa, Shabir Lakhi, Suilanji Sivile, Mallika Yavatkar, Brianna Lindsay, Kristen Stafford, Lottie Hachaambwa, Lloyd Mulenga, Cassidy W. Claassen

**Affiliations:** 1 Adult Infectious Diseases Center, University Teaching Hospital, Lusaka, Zambia; 2 University of Alabama at Birmingham School of Medicine, Birmingham, AL USA; 3 University of Maryland School of Medicine, Baltimore, MD USA; 4 Division of Infectious Diseases, Institute of Human Virology, University of Maryland School of Medicine, Baltimore, MD USA; 5 Center for International Health, Education, and Biosecurity, University of Maryland School of Medicine, Baltimore, MD USA; 6 Division of Infectious Diseases, Department of Medicine, Vanderbilt University Medical Center (VUMC), Nashville, TN USA; 7 Vanderbilt Institute for Global Health (VIGH), Nashville, TN USA; 8 Ministry of Health, Ndeke House, Lusaka, Zambia

**Keywords:** Advanced HIV disease, TB diagnostics, Viral load monitoring, Sub-Saharan Africa

## Abstract

**Background:**

Zambia recently achieved UNAIDS 90-90-90 treatment targets for HIV epidemic control; however, inpatient facilities continue to face a large burden of patients with advanced HIV disease and HIV-related mortality. Management of advanced HIV disease, following guidelines from outpatient settings, may be more difficult within complex inpatient settings. We evaluated adherence to HIV guidelines during hospitalization, including opportunistic infection (OI) screening, treatment, and prophylaxis.

**Methods:**

We reviewed inpatient medical records of people living with HIV (PLHIV) admitted to the University Teaching Hospital in Lusaka, Zambia between December 1, 2018 and April 30, 2019. We collected data on patient demographics, antiretroviral therapy (ART), HIV biomarkers, and OI screening and treatment—including tuberculosis (TB), Cryptococcus, and OI prophylaxis with co-trimoxazole (CTX). Screening and treatment cascades were constructed based on the 2017 WHO Advanced HIV Guidelines.

**Results:**

We reviewed files from 200 charts of patients with advanced HIV disease; of these 92% (184/200) had been on ART previously; 58.1% (107/184) for more than 12 months. HIV viral load (VL) testing was uncommon but half of VL results were high. 39% (77/200) of patients had a documented CD4 count result. Of the 172 patients not on anti-TB treatment (ATT) on admission, TB diagnostic tests (either sputum Xpert MTB/RIF MTB/RIF or urine TB-LAM) were requested for 105 (61%) and resulted for 60 of the 105 (57%). Nine of the 14 patients (64%) with a positive lab result for TB died before results were available. Testing for Cryptococcosis was performed predominantly in patients with symptoms of meningitis. Urine TB-LAM testing was rarely performed.

**Conclusions:**

At a referral hospital in Zambia, CD4 testing was inconsistent due to laboratory challenges and this reduced recognition of AHD and implementation of AHD guidelines. HIV programs can potentially reduce mortality and identify PLHIV with retention and adherence issues through strengthening inpatient activities, including reflex VL testing, TB-LAM and serum CrAg during hospitalization.

## Introduction

Global scale up of antiretroviral therapy (ART) has attained high coverage levels and prevented over 12.1 million HIV-related deaths worldwide, yet many clients still present with advanced HIV disease [1–4]. Declines in HIV-related mortality and morbidity observed between 2003 and 2015 have plateaued, particularly in sub-Saharan Africa [5, 6]. In Zambia, mortality among people living with HIV (PLHIV) following linkage to care remains high, at 10.3 per 100 person-years among men with at least 18 months of ART (compared to 5.5 per 100 person years among women) which is slightly higher than the 6.7 and 6.9 per 100 person years reported in South Africa and Ethiopia respectively [7–9]. Additionally, post-discharge mortality among PLHIV was found to be 22% at the largest hospital in Zambia [2]. While there is growing interest in non-communicable diseases among PLHIV [4], it is likely that HIV-associated mortality is partly driven by the prevalence of advanced HIV disease, defined as HIV infection with CD4 count < 200 cells/mm^3^ or WHO clinical stage 3 or 4 disease [10].

Guidelines were developed by the World Health Organization (WHO) for the detection and management of advanced HIV in 2017, based on randomized controlled trials and expert opinion [10–12]. These guidelines emphasize the need for CD4 count testing and diagnosis, treatment, and prevention of opportunistic infections (OIs) such as tuberculosis and cryptococcosis, the OIs that are responsible for a large proportion of HIV-related morbidity and mortality [10]. It is necessary to establish the effectiveness of these care packages in treatment-experienced patients in various clinical settings [11].

Almost two decades into the widespread availability of ART in Lower-Middle Income Countries (LMIC), most PLHIV are treatment experienced, and the guidelines have expanded to include ART monitoring [10]. However, much of the data and expert opinion underpinning these guidelines were derived in outpatient models of chronic care and several landmark clinical trials that enrolled ART-naïve outpatients, the majority of whom had asymptomatic HIV [13–16]. Compared to outpatient and community programs, inpatient settings are more focused on immediate life-saving interventions, which may result in missed opportunities to recognize and address advanced disease among PLHIV.

We evaluated the implementation of WHO Advanced HIV Disease Guidelines at the University Teaching Hospital (UTH) in Lusaka, Zambia, a setting with high inpatient HIV prevalence. We sought to identify gaps in requesting and providing recommended tests and treatment and explored timeliness of screening tests at UTH. The purpose of our study was to describe current practices and identify opportunities to reduce the impact of advanced disease on HIV-related mortality.

## Methods

### Study setting

The UTH is a 1665-bed capacity hospital and the largest referral center in the country. The six inpatient Internal Medicine wards—collectively known as E-block—are reserved for patients cared for by various Internal Medicine subspecialties. HIV rapid testing is opt-out among all admitted patients who report being HIV negative or unknown status. Requests for laboratory tests other than HIV rapid testing are documented by clinicians in the patient file. Samples are collected on the wards and taken to the central laboratories, where test results are entered into a laboratory information system (LIS). The urine lipoaribomannan assay for tuberculosis (TB-LAM), which can be performed at the bedside, is documented in the paper file. Once LIS reports results, they are printed for filing in the patient chart. All prescribed medications are listed in a paper tool that accompanies the chart. We performed a retrospective chart review of services provided between 1st December 2018 and 30th April 2019 using inpatient records at the end of hospitalization (discharge or death).

### Study procedures

We identified eligible patients using ward registers, which are the most comprehensive tool available to track E-block admissions. Inclusion criteria was age 16 years or older, HIV infection (based on documentation of self-reported HIV positive or a positive test in the file), and admission to the medical wards under the care of the Infectious Diseases Unit. Patients who left the hospital against medical advice, were excluded from the study. Patients were also excluded if files were inaccessible post discharge; this most often occurred because they opted for post discharge review at their local clinic and not at the UTH. Using a standardized tool, we extracted patient demographic data, presenting symptoms, laboratory testing including HIV biomarkers (CD4 count and VL) and OI screening using the LIS, and pharmacy records related to OI treatment and prevention.

Our assessment focused on four domains adapted from the 2017 WHO Advanced HIV Guidelines [10]. First, we assessed ART monitoring as guidelines recommend VL testing in treatment-experienced patients at six months post initiation, then annually if the patient is virologically suppressed and deemed stable. CD4 testing is to be done at baseline then every six months thereafter [10]. Second, we assessed the proportion of those prescribed OI prophylaxis, as patients with CD4 counts less than 350 cells/mm^3^ should be commenced on cotrimoxazole (CTX) prophylaxis. Third, we evaluated tuberculosis (TB) care at UTH. Per guidelines, all patients should be assessed for TB via the WHO TB screening questions, a urine TB-LAM, and/or Gene Xpert MTB/RIF testing. Those who test negative for TB are to receive six months of isoniazid as TB preventative therapy (TPT). Finally, we assessed Cryptococcosis management. For CD4 counts less than 100 cells/mm^3^ or with symptoms such as headache and fever suggestive of cryptococcal meningitis, serum Cryptococcal antigen (CrAg) test is warranted and fluconazole prophylaxis until the CD4 count is greater than 350 cells/mm^3^.

### Statistical analysis

Microsoft Excel version 16 (Microsoft Corporation, Seattle, USA) was used to store raw data and R version 4.0.3 (R Core Team, 2021) was used for analyses. We described patient characteristics and pre-hospital use of ART and anti-tuberculous therapy (ATT) on admission to the hospital. Then, we constructed four ‘care cascades’ as follows: (a) ART monitoring with HIV VL, (b) appropriate use of CTX based on CD4 count < 350 cells/mm^3^, (c) TB testing and treatment, and (d) cryptococcosis diagnostic testing. These cascades were guided by the 2017 WHO Advanced HIV disease guidelines [10], which were incorporated in the Zambia Consolidated Treatment Guidelines as early as 2018 [17]. When possible, we also described the timing of test resulting in relation to the admission course. Descriptive statistics were used to analyze baseline characteristics. Continuous variables were expressed as median and interquartile range (IQR) whilst categorical variables were expressed as a percentage from the group which they were derived.

### Ethical approvals

The study was approved by UTH, the University of Zambia Biomedical Research Ethics Committee (Ref. 012-02-19), the University of Maryland Baltimore institutional review board (IRB), and the Zambia National Health Research Authority (Ref No. NHRA 2019-05-21). Given the retrospective nature of the study, a waiver of informed consent was obtained from the IRBs and no patient identifiers were collected.

## Results

### Baseline characteristics

Between 1st December 2018 to 1st May 2019, there were 331 individuals eligible for chart review and 200 were enrolled in the study. We excluded 11 who left against medical advice and 120 who were discharged but did not leave their chart behind against hospital policy. Patient characteristics are reported in Table 1. Among included participants, the average length of stay was 11 days, with a minimum of 1 day and a max of 56 days. A total of 109 (54%) of participants were female. Median age was 39 years with 76% below 50 years of age [IQR 31–45 years]. CD4 count results were frequently unavailable (57% missing), but of those with available data, 62% of patients had advanced HIV disease (48 patients of the 77 patients with available CD4 results). There was no difference in the percent of patients with documented CD4 cell count among the patients who died compared to those who were discharged (28% and 22% respectively).

**Table 1:** Demographics and baseline characteristics of patients

	All patients N = 200
Sex (n [%])	
Female	109 [54.5]
Male	91 [45.5]
Patient Primary Complaint (n reporting [%] for each)	
Cough, fevers, or night sweats	52 [26.0]
Diarrhea, vomiting or abdominal pains	42 [21.0]
Headache, seizures or altered mental status	37 [18.5]
Weakness or palpitations	21 [10.5]
Difficulty breathing and other respiratory complaints	8 [4.0]
Renal dysfunction	8 [4.0]
Other Conditions†	32 [16.0]
Duration of hospital stay (median [ IQR] days)	7 [5–10]
Patient age (median [IQR] years)	39 [31–45]
ART status on admission (n [%])	
Treatment experienced	184 [92.0]
Duration on ART > 12 months	107 [58.1]
Disengaged from care	23 [13]

^†^Other Conditions: Caustic ingestion, jaundice, chronic flank pain, Severe malaria, lupus, Cerebral Vascular Accidents, poisoning, dysphagia, Kaposi sarcoma, limb pain, eye pain, epileptic seizures, swollen testicles, molluscum contagiosum, infected decubitus ulcers, dermatopathology, pelvic mass

### Inpatient advanced HIV diagnostic and treatment cascades

**Fig. 1:**
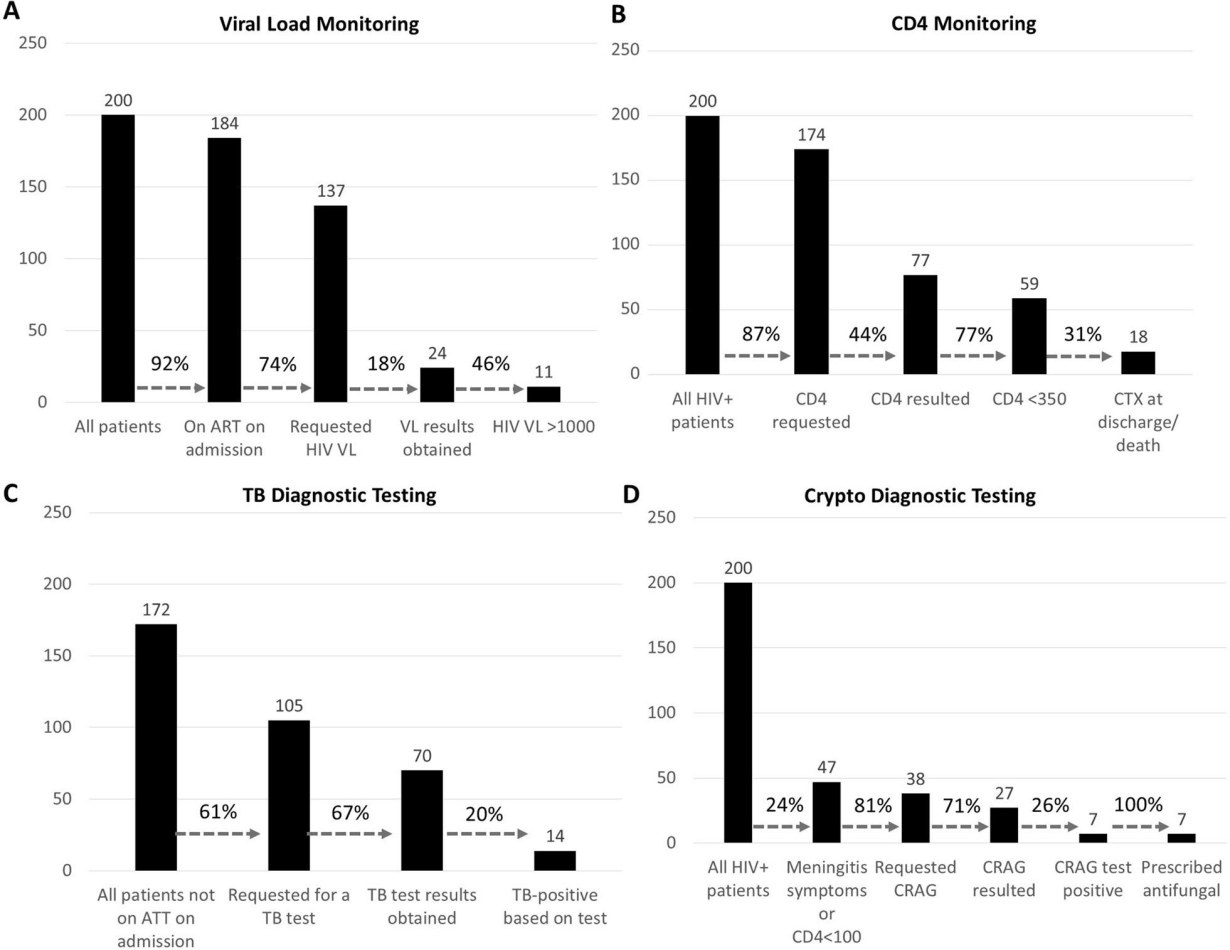
Advanced HIV disease testing cascades. **A** Number of patients receiving viral load monitoring. **B** Number of patients receiving monitoring of CD4 count. **C** Number of patients receiving TB Diagnostic testing. **D** Number of patients undergoing cryptococcal diagnostics.

Of the 200 patients in this study, 92% were treatment experienced at the time of admission, including 13% who needed to reengage into care (23/184). Clinicians requested HIV VL for 146 patients yet only 26 (18%) had results available at the end of inpatient care (Fig. 1A). When results were available, over half (14 of 26, 54%) had HIV VL > 1000copies/ml (median 5.5 Log [IQR 5.3–6.1 Log]; suggesting ART failure or disengagement from care. Among the 184 ART-experienced patients, 33 (18%) were switched during hospitalization to either second-line therapy or for reasons of renal or hepatic dysfunction.

Figure 1B shows monitoring of CD4 counts and appropriate use of CTX at discharge. Among participants reviewed, a clinician requested CD4 count for 87%; however, results were documented in only 44% (77 of 174), on average on the 5th day of admission. When resulted, CD4 counts were < 350 cells/mm^3^, the threshold for CTX as OI prophylaxis, 77% of the time (59 of 77). There was evidence of CTX prescription (on admission, during admission, or at discharge) for few participants (18 of 200 participants), and only 31% of those with CD4 count < 350 cells/mm^3^ (18 of the 59).

Among participants, 28 were on ATT on admission, (Fig. 1C). Of the remaining 172, 107 (62%) were assessed for TB. Assessment included screening for typical TB symptoms in 43 patients (40%) i.e., cough, fever, weight loss or night sweats. By discharge or death, 36 patients had newly initiated ATT, including 4 with a positive result and an additional 32 in whom there was high clinical suspicion. Nine of the 14 patients (64%) with a positive result had died before the results were available.

WHO criteria for CrAg testing were met by 24% of patients (47 of the 200 patients), i.e., 18 with symptoms suggestive of meningitis and an additional 29 presented with CD4 counts less than 100 cells/mm^3^ (Fig. 1D) [10]. Serum CrAg testing was not routinely available, and 38 of the 47 eligible (81%) accepted to have their CSF tested. Six of the seven patients with a positive CrAg were discharged on fluconazole, one had died whilst receiving fluconazole, and two additional patients received fluconazole prophylactically.

Across all diagnostic tests, we also assessed the time from test resulting in LIS to filing in the chart. Over 95% of results in the computer and file were concordant. The time from resulting in the LIS system and filing was < 1 day on average (median 3 days [IQR 1–6 days]).

## Discussion

We conducted a retrospective, descriptive study for the management of patients with advanced HIV disease compared to guideline recommendations to identify significant gaps in advanced HIV care at a large volume inpatient tertiary setting in Zambia where HIV burden is high. This study seeks to build on the growing evidence examining the effectiveness of the AHD packages for treatment-experienced patients in SSA [11, 18]. We highlight the risk of adverse patient outcome and sustained mortality in patients with AHD when appropriate screening and pre-emptive treatments are not expedited [18].

Opportunistic infections were likely under-ascertained in part due to incomplete recognition of the syndrome of advanced HIV, as well as challenges in laboratory diagnosis [19–21]. Meya and colleagues proposed indicators for advanced HIV and called for strengthened rapid laboratory diagnostics, such as LAM and CrAg, for hospitalized patients to close gaps similar to those seen in our study [18]. Hospitalization presents a good opportunity to identify AHD and offer bundled therapy to reduce HIV-associated mortality and morbidity because these settings have stronger capacity and time to implement care (as patient is admitted) [1, 2]. However, outpatient approaches to AHD are essential to reduce admissions.

Data from this study also shows that inpatient settings should be exploited to identify ART treatment failure. In our patient population, 50% of PLHIV were not virally suppressed, compared to 10–15% non-suppression nationally [22]. This suggests that universal VL testing on hospitalization would likely identify a larger number of non-suppressed patients, which would then prompt early intervention by clinicians. At present, a major challenge to universal inpatient VL testing in LMICs may be the costs of reagents and consumables [23]. As outpatient records, which could include a recent VL result, are often not available at admission, inpatient VLs testing may be viewed as wasteful in these settings. Another challenge to implementing inpatient VL testing is the lack of data on clinician use of results. In higher income countries clinicians often fail to routinely utilize VL results available to optimize patient regimens [6, 24, 25]. Similar data are lacking in LMIC settings.

In settings with high advanced HIV burden, TB-LAM and CrAg could be performed on all PLHIV admitted to the hospital. These tests can be sent even before CD4 count results are available, to shorten the time lapse of rapid tests that can be performed at bedside, especially if lab reagent supply constraints limit CD4 testing. We found that lack of CD4 testing was associated with an inordinate lapse in the number of clients receiving CTX prophylaxis. This downward trend in CD4 testing has been demonstrated across the southern Africa region programmatically and may result in missed diagnoses of advanced HIV disease, thus threatening the effectiveness of ART programs [6].

The limitations of this study are largely attributable to the retrospective study design, which has a greater propensity for missing data, and selection bias may have been introduced due to the missing files from the patients who left with their files for review at their primary health centers as opposed to the UTH. Additionally, the facility presently does not have an electronic health record system which would contribute to availability of patient files and reporting of lab results. Whilst nearly all PLHIV were ART-experienced, it is difficult to conclude what proportion of these clients required additional VL testing without access to the outpatient care file.

Strengths include that the study was done at the largest tertiary center in the country with the largest resource allocation and the use of the LIS, which allowed us to accurately measure test resulting in the hospital.

## Conclusions

At a referral hospital in Zambia, CD4 testing was inconsistent due to laboratory challenges and this reduced recognition of AHD and implementation of AHD guidelines. HIV programs can potentially reduce mortality and identify PLHIV with retention and adherence issues through strengthening inpatient activities, including reflex VL testing, TB-LAM and serum CrAg during hospitalization.

## Data Availability

All datasets generated or analysed in this current study are available from the corresponding author on reasonable request.

## Abbreviations

AHD: Advanced HIV disease
ART: Antiretroviral therapy
ATT: Antituberculosis therapy
CrAg: Cryptococcal antigen test
CSF: Cerebral spinal fluid
CTX: Co-trimoxazole
HIV: Human immunodeficiency virus
IQR: Interquartile range
LIS: Laboratory information system
LMIC: Lower-middle income countries
OIs: Opportunistic infections
PLHIV: People living with HIV
SD: Standard deviation
TB: Tuberculosis
TB-LAM: Lateral flow urine lipoaribomannan assay for tuberculosis
TPT: TB Preventative therapy
VL: Viral load test
UTH: University Teaching Hospital
WHO: World Health Organization
ZamPHIA: Zambia Population -based HIV Impact Assessment

## References

[1] Carmona S, Bor J, Nattey C, Maughan-Brown B, Maskew M, Fox MP (2018). Persistent high burden of advanced HIV disease among patients seeking care in South Africa’s National HIV Program: data from a Nationwide Laboratory Cohort. Clin Infect Dis.

[2] Haachambwa L, Kandiwo N, Zulu PM, Rutagwera D, Geng E, Holmes CB (2019). Care continuum and postdischarge outcomes among HIV-infected adults admitted to the hospital in Zambia. Open Forum Infect Dis.

[3] Lebelonyane R, Mills LA, Mogorosi C, Ussery F, Marukutira T, Theu J (2020). Advanced HIV disease in the Botswana combination prevention project: prevalence, risk factors, and outcomes. AIDS.

[4] UNAIDS. UNAIDS State of the epidemic. UNAIDS 2018 Reference. 2018.

[5] Jahagirdar D, Walters MK, Novotney A, Brewer ED, Frank TD, Carter A (2021). Global, regional, and national sex-specific burden and control of the HIV epidemic, 1990–2019, for 204 countries and territories: the Global Burden of Diseases Study 2019. Lancet HIV.

[6] Zaniewski E, Dao Ostinelli CH, Chammartin F, Maxwell N, Davies MA, Euvrard J (2020). Trends in CD4 and viral load testing 2005 to 2018: multi-cohort study of people living with HIV in Southern Africa. J Int AIDS Soc.

[7] Kerkhoff AD, Sikombe K, Eshun-Wilson I, Sikazwe I, Glidden D V., Pry JM, et al. Mortality estimates by age and sex among persons living with HIV after ART initiation in Zambia using electronic medical records supplemented with tracing a sample of lost patients: a cohort study. PLoS Med. 2020;17(5). 10.1371/journal.pmed.1003107 PMC7219718 32401797

[8] Workie KL, Birhan TY, Angaw DA. Predictors of mortality rate among adult HIV-positive patients on antiretroviral therapy in Metema Hospital, Northwest Ethiopia: a retrospective follow-up study. AIDS Res Ther. 2021;18(1). 10.1186/s12981-021-00353-z PMC8097881 33952282

[9] Dawood H, Hassan-Moosa R, Zuma NY, Naidoo K. Mortality and treatment response amongst HIV-infected patients 50 years and older accessing antiretroviral services in South Africa. BMC Infect Dis. 2018;18(1). 10.1186/s12879-018-3083-z PMC5894176 29636023

[10] World Health Organization. Guidelines for managing advanced HIV disease and rapid initiation of antiretroviral therapy. Licence: CC BY-NC-SA 3.0 IGO. [Internet]. 2017. 1–56 p. Available from: http://apps.who.int/iris/bitstream/10665/255884/1/9789241550062-eng.pdf?ua=1. 29341560

[11] Ford N, Meintjes G, Calmy A, Bygrave H, Migone C, Vitoria M (2018). Managing advanced HIV disease in a public health approach. Clin Infect Dis.

[12] MOH GRZ. Guidelines for Treatment and Prevention of HIV Infection. 2020.

[13] Mfinanga S, Chanda D, Kivuyo SL, Guinness L, Bottomley C, Simms V (2015). Cryptococcal meningitis screening and community-based early adherence support in people with advanced HIV infection starting antiretroviral therapy in Tanzania and Zambia: an open-label, randomised controlled trial. Lancet.

[14] Manosuthi W, Wiboonchutikul S, Sungkanuparph S (2016). Integrated therapy for HIV and tuberculosis. AIDS Res Ther.

[15] Hakim J, Musiime V, Szubert AJ, Mallewa J, Siika A, Agutu C (2017). Enhanced prophylaxis plus antiretroviral therapy for advanced HIV infection in Africa. N Engl J Med.

[16] Cohen MS, Chen YQ, McCauley M, Gamble T, Hosseinipour MC, Kumarasamy N (2016). Antiretroviral therapy for the prevention of HIV-1 transmission. N Engl J Med [Internet]..

[17] MOH GRZ. Zambia consolidated guidelines for Treatment and Prevention of HIV infection. 2018.

[18] Meya DB. Establishing targets for advanced HIV disease: a call to action. 1–5. 10.4102/sajhivmed.v22i1.1266 PMC8424734 34522428

[19] Andrews B, Semler MW, Muchemwa L, Kelly P, Lakhi S, Heimburger DC (2017). Effect of an early resuscitation protocol on in-hospital mortality among adults with sepsis and hypotension: a randomized clinical trial. JAMA J Am Med Assoc.

[20] Barr DA, Lewis JM, Feasey N, Schutz C, Kerkhoff AD, Jacob ST, et al. Mycobacterium tuberculosis bloodstream infection prevalence, diagnosis, and mortality risk in seriously ill adults with HIV: a systematic review and meta-analysis of individual patient data. Lancet Infect Dis. 2020;1–11. 10.1016/S1473-3099(19)30695-4 PMC7254058 32178764

[21] Molloy SF, Kanyama C, Heyderman RS, Loyse A, Kouanfack C, Chanda D (2018). Antifungal combinations for treatment of cryptococcal meningitis in Africa. N Engl J Med.

[22] Global Aids Monitoring 2019. Country progress report—Zambia. 2019;

[23] Craik A, Patel P, Patel P, Mallewa J, Malisita K, Bitilinyu-Bangoh J (2016). Challenges with targeted viral load testing for medical inpatients at Queen Elizabeth Central Hospital in Blantyre, Malawi. Malawi Med J.

[24] Roberts T, Cohn J, Bonner K, Hargreaves S (2016). Scale-up of routine viral load testing in resource-poor settings: current and future implementation challenges. Clin Infect Dis.

[25] Raizes E, Hader S, Birx D (2018). The US President’s Emergency Plan for AIDS relief (PEPFAR) and HIV drug resistance: mitigating risk. Monit Impact.

